# Analysis of medical service utilization for post-stroke sequelae in Korea between 2016 and 2018: a cross-sectional study

**DOI:** 10.1038/s41598-022-24710-8

**Published:** 2022-11-28

**Authors:** Hyun-Jun Lee, Yu-Cheol Lim, Ye-Seul Lee, Seungwon Kwon, Yoon Jae Lee, In-Hyuk Ha

**Affiliations:** 1 Jaseng Hospital of Korean Medicine, 536 Gangnam-daero, Gangnam-gu, Seoul, 06110 Republic of Korea; 2 Jaseng Spine and Joint Research Institute, Jaseng Medical Foundation, 2F, 540 Gangnam-daero, Gangnam-gu, Seoul, 06110 Republic of Korea; 3 Department of Cardiology and Neurology, College of Korean Medicine, Kyung Hee University, Seoul, 02447 Republic of Korea

**Keywords:** Health care economics, Health services, Cerebrovascular disorders

## Abstract

In this retrospective cross-sectional observational study, the medical service utilization of post-stroke sequelae patients was examined using a national patient sample. The Korean Health Insurance Review and Assessment Service-National Patients Sample database was used to investigate the medical service utilization of 19,562 patients, diagnosed with post-stroke sequelae of cerebrovascular disease (I69) in Korea between January 2016 and December 2018. We compared the demographic characteristics, diagnosis code subtypes, frequency of healthcare utilization, medical costs, and comorbidities of standard care (SC) and Korean medicine (KM) users. Overall, patients aged ≥ 65 years accounted for the highest percentage, and utilization of medical services increased among patients aged ≥ 45 years. Outpatient care was higher among SC (79.23%) and KM (99.38%) users. Sequelae of cerebral infarction accounted for the highest percentage of diagnosis subtypes. Physical therapy and rehabilitation therapy were most frequent in SC, whereas injection/procedure and acupuncture were most frequent in KM. Cerebrovascular circulation/dementia drugs were prescribed most frequently in SC. Circulatory, digestive, endocrine, and metabolic disorders were the most common comorbidities in SC, whereas musculoskeletal and connective tissue disorders were most common in KM. Overall, SC and KM users showed differences in the number of medical service claims, cost of care, and comorbidities. Our findings provide basic research data for clinicians, researchers, and policy makers.

## Introduction

Post-stroke sequelae refer to the medical, musculoskeletal, and psychosocial symptoms and additional disabilities that appear with the various clinical outcomes of stroke^1^. Stroke can cause sequelae that are accompanied by serious long-term disability^2^. According to one study, 40% of stroke survivors experience sequelae within one month to five years^3^. The short-term clinical symptoms of post-stroke sequelae include urinary infection, aspiration pneumonia, decubitus ulcers, and constipation, and the long-term ones include pain syndromes, depression, anxiety, cognitive impairment, dementia, epilepsy, and gait instability^4^. Other major sequelae include neurological deficits, conscious disturbance, cognitive and behavioral changes, paralysis, dysphagia, and aphasia^5^. Of these, sequelae that include conditions specified as late effects or those present one year or more after the onset of the causal condition are classified as I69 Sequelae of cerebrovascular disease by the International Classification of Disease and Cause of Death-10 (ICD-10).

The prevalence of stroke is increasing; however, its related mortality rate is decreasing^4^. As a result, the prevalence of post-stroke sequelae seems to be increasing^5,6^. In the US, the prevalence of stroke was estimated to be 2.5% between 2013 and 2016, and was shown to increase with an aging population. In contrast, the mortality rate of stroke decreased by 16.7% between 2006 and 2016. Globally, the prevalence of ischemic stroke increased by 2.7% between 2006 and 2016, whereas the mortality rate of cerebrovascular disease decreased by 21% over the same period^4^.

Treatments for post-stroke sequelae are aimed at various symptoms. Standard care (SC) involves the use of pharmacological treatment (using drugs with various mechanisms), physical therapy, rehabilitation treatment, psychotherapy, and injection therapy. Pharmacological treatments and individualized physical exercise programs are used for symptoms of general pain and stiffness due to post-stroke sequelae^7,8^. Central post-stroke pain is treated with pharmacological treatment using antidepressants (amitriptyline), anticonvulsants (gabapentin and pregabalin), opioids, and morphine, as well as motor cortex stimulation^9,10^. For neuropsychiatric sequelae, selective serotonin reuptake inhibitors are used to improve motor outcome, whereas depression is treated with psychotherapy, including the use of antidepressants and neurostimulation^11^. Dysphagia is treated with nerve and electrical stimulation therapy (e.g., pharyngeal electrical stimulation, neuromuscular electrical stimulation, repetitive transcranial magnetic stimulation, and transcranial direct current stimulation) and surface electromyography (sEMG)^12^.

South Korea has a dichotomized healthcare system that allows its citizens to receive both SC and Korean medicine (KM) treatments, which are covered by the national health insurance (NHI)^13,14^. KM involves the use of acupuncture, moxibustion, electroacupuncture (transcutaneous electrical nerve stimulation [TENS] on acupuncture), herbal medicine^15^, and cupping^16^. Acupuncture is used to improve dependency in daily activities and to treat neurological deficiencies and various functional impairments that appear as post-stroke sequelae. It is also used to treat cognitive impairment, dysphagia, depression, anxiety, and general pain (including shoulder pain)^17,18^, as well as for improving post-stroke spastic hemiplegia (PSSH) and range of motion (ROM)^19^. Electroacupuncture is used for the recovery of overall function after a stroke, to improve spasticity and motor impairment, and to treat reflex sympathetic dystrophy^20–22^. Moxibustion is used to treat PSSH, urinary incontinence, and constipation^23–25^. Herbal medicine is used for the post-stroke recovery of neurological functional deficits^26^, as well as for treating post-stroke anxiety, depression, dyspepsia, spasticity, and fatigue^27–30^.

Previous studies have used domestic data to analyze the physical activity and health-related quality of life according to the gender of stroke survivors^31,32^. One study analyzed the association between stroke status and post-stroke depression^33^, and other studies have analyzed cognitive impairment and the association between post-stroke spasticity and functional impairments^34–36^. Studies on stroke-related diseases have investigated the characteristics, treatment, management, and disability-adjusted life years lost^37–39^ of these diseases, and there have also been epidemiological reports from the Korean Stroke Society and Clinical Research Center for Stroke^40,41^. Furthermore, various studies have analyzed the associations between stroke and various factors such as blood lead level, open-angle glaucoma, and metabolic obesity vs. non-obese weight^42–44^. However, there is little information on the latest medical services being provided in clinical practice for the treatment of post-stroke sequelae.

All claims filed with the NHI are archived as data that can be used to easily identify the clinical status of any specific disease code. Nevertheless, few studies have used real-world data to examine the status of medical services for post-stroke sequelae. In addition, studies that include both SC and KM treatments are even scarcer. With the forecast of a gradual increase in disease burden and the direct/indirect costs associated with post-stroke sequelae, additional studies regarding the reality of medical services are needed. In this study, we used 3-year NHI data to investigate the utilization of medical services related to post-stroke sequelae—an analysis that was lacking in most previous studies—and present the current status of these services. Our findings comprehensively reflect the current clinical practice of treatment for post-stroke sequelae, and we include an analysis of data related to KM treatments.

## Methods

### Data source

We used national patient sample (NPS) data (updated annually by the Health Insurance Review and Assessment Service [HIRA]) over the period of January 1, 2016, to December 31, 2018. These data were collected by the HIRA by sampling 3% of all patients who had received treatment under the NHI scheme (approximately 1,400,000 patients). The data—consisting of stratified random samples based on claims data—are secondary data statistically sampled from raw data after removing personal or corporate information^45^. The data included information regarding the sociodemographic characteristics of patients (including gender, age, and medical aid program), the primary diagnoses, sub-diagnoses, medical services received, medical institutions visited, and healthcare costs, including co-payments. However, this data does not cover non-reimbursement services and the usage of over-the-counter medicines.

### Study design and study population

To retrieve information regarding patients with post-stroke sequelae from the HIRA-NPS database, we used the seventh revision of the Korean Standard Classification of Diseases (KCD-7) diagnosis code I69 (Sequelae of cerebrovascular disease). The KCD-7 reflects the International Classification of Disease and Cause of Death-10 (ICD-10). Category I69 indicates the conditions in I60–I67.1 and I67.4–I67.9 as the causes of sequelae, which are classified elsewhere. The sequelae include the conditions specified as such or as late effects, or those present one year or more after the onset of the causal condition. Category I69 Sequelae of cerebrovascular disease is subdivided into I69.0 Sequelae of subarachnoid hemorrhage; I69.1 Sequelae of intracerebral hemorrhage; I69.2 Sequelae of other nontraumatic intracranial hemorrhage; I69.3 Sequelae of cerebral infarction; I69.4 Sequelae of stroke, not specified as hemorrhage or infarction; and I69.8 Sequelae of other and unspecified cerebrovascular diseases.

We retrieved the data of patients with records that included diagnosis code I69 as the main diagnosis or sub-diagnosis between January 1, 2016, and December 31, 2018, and used the data of patients with significant sequelae. The following records were excluded: form code indicating dentistry, health center, or psychiatry; type of institution being a psychiatric hospital, dental hospital, postpartum care facility, or health center; and total expense or number of days in care being 0 or blank.

### Study outcomes

In the present study, the baseline demographic characteristics of the patients were summarized by age, gender, payer type, type of visit, and type of medical institution. Moreover, the patients (aged < 15 to ≥ 75 years) were divided into eight age groups in 10-year increments. Payer type was divided into NHI, Medicaid, and others. Age, gender, and payer type were further divided based on the number of patients. The type of visit was divided into outpatient and inpatient care. The type of medical institution was divided into tertiary hospital/general hospital/hospital, clinic, convalescent hospital, KM hospital, and KM clinic. These were further divided based on the number of records. These baseline demographic characteristics were analyzed separately for SC users and KM users for all categories. SC institutions included tertiary hospital/general hospital/hospital, clinic, and convalescent hospital, while KM institutions included KM hospital and KM clinic.

The subtypes of diagnosis code I69 were analyzed based on the number of claims for each year. The subtypes I69.0 Sequelae of subarachnoid hemorrhage, I69.1 Sequelae of intracerebral hemorrhage, and I69.2 Sequelae of other nontraumatic intracranial hemorrhage were categorized as “hemorrhage”; I69.3 Sequelae of cerebral infarction was categorized as “infarction”; I69.4 Sequelae of stroke, not specified as hemorrhage or infarction was categorized as “others”; and I69.8 Sequelae of other and unspecified cerebrovascular diseases was categorized as “unspecified.” Because I69 Sequelae of cerebrovascular disease accounted for only ~ 0.1% of sequelae, it was not included in the graph.

Total healthcare costs—including those for both inpatient and outpatient care—were categorized according to the medical services provided (excluding medication, as recorded in the claims data). The number of claims and 3-year healthcare costs for each category were divided into SC and KM groups for comparison. Additionally, all claims records were categorized based on the most frequently claimed service codes for comparison between the SC and KM groups. The total claims, costs per claim, and costs per patient during 2016–2018 were analyzed separately for each year. For SC, the categories included consultation fee, rehabilitation treatment, heat therapy, injection therapy, physical therapy, testing fee, treatment fee, and prescription/medicine fee, among others. For KM, the categories included consultation fee, acupuncture, moxibustion, cupping, thermotherapy and cryotherapy of the meridian, electroacupuncture stimulation, and prescription/medicine fee, among others. For medication, each drug was categorized according to the Anatomical Therapeutic Chemical classification code in accordance with the classification standards of the Ministry of Health Welfare. The frequency and cost of prescribed drugs were analyzed by category. To analyze comorbidities associated with post-stroke sequelae, we identified other diagnosis codes that included diagnosis code I69, and then divided these into SC and KM groups for comparison.

### Statistical analysis

We performed a descriptive analysis in this study to summarize the study population data (e.g., frequency, percentage, and means). Each category's average log change was presented as an annual change rate. The change rate was calculated assuming that the frequency of each item increased at a constant rate over time. Subtypes of sequelae of cerebrovascular disease of each patient between years were tested using the Chi-square test. The annual trend of the utilization of service categories was statistically tested using univariate linear regression. The calendar year of the claims was the independent variable, and the annual number of claims, claims per patient, and cost per patient of each service category were the dependent variables in the regression model, respectively. All costs were converted using the currency exchange rate (KRW to USD) for the corresponding year and adjusted according to the health sector consumer price index for 2018. The data were analyzed using the SAS software (version 9.4, SAS Institute, Cary, NC, USA).

### Ethics approval

The study protocol was approved by the HIRA Deliberative Committee for public data provision, and the study was conducted in accordance with relevant guidelines and regulations. The current study was reviewed and qualified for an exemption by the Institutional Review Board of Jaseng Hospital of Korean Medicine, Seoul, Korea (2021-02-028). Because the study analyzed publicly available data, no consent was obtained from the subjects by the authors. Moreover, all personal information was de-identified by the National Health Insurance Service (NHIS) prior to public release. The principles expressed in the Declaration of Helsinki have been adhered to in the analysis.

## Results

### Baseline characteristics of patients

**Figure 1:**
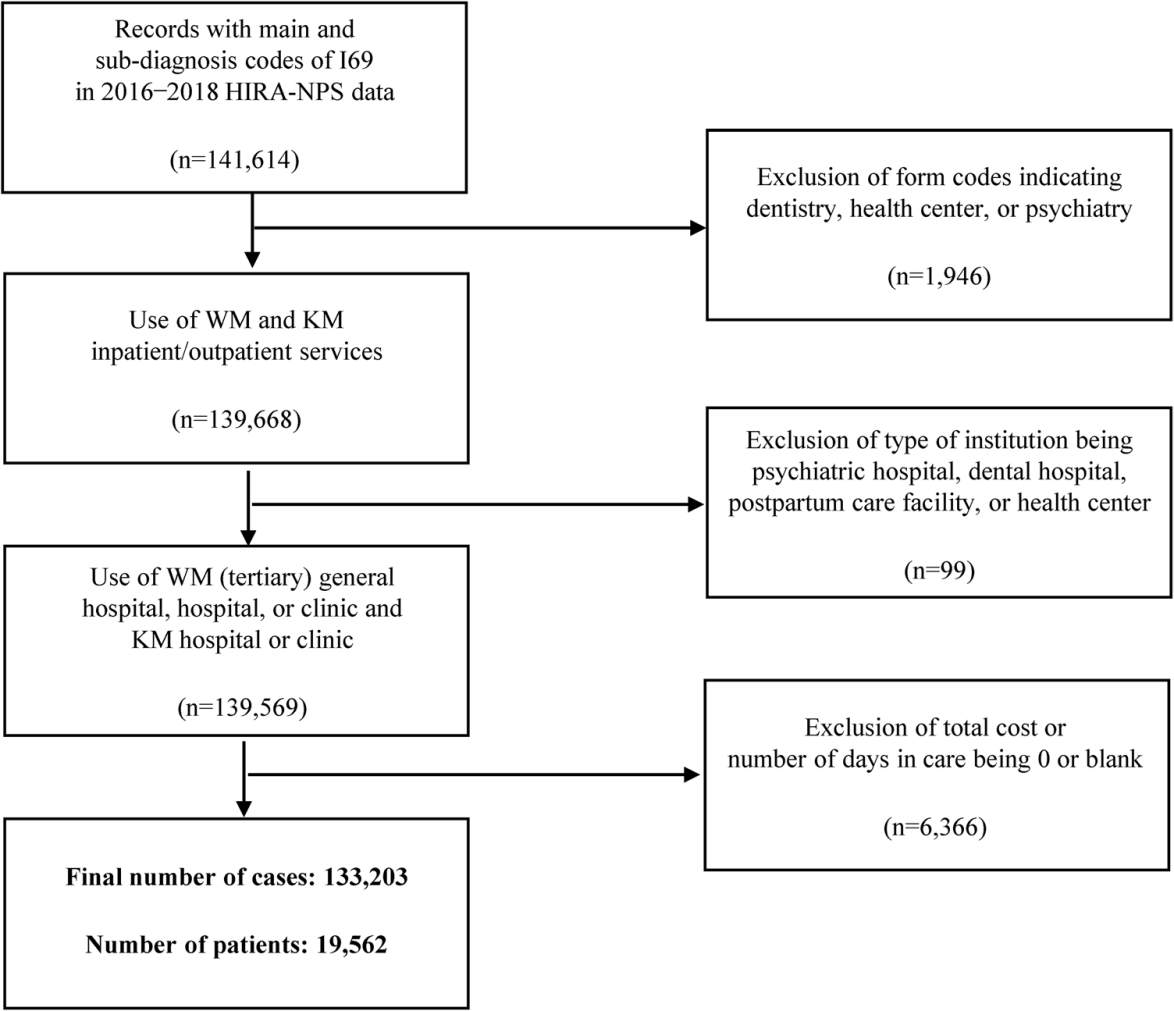
Flowchart of the study design. HIRA-NPS: Health Insurance Review and Assessment Service-National Patients Sample; SC: Standard care; KM: Korean medicine.

Between January 1, 2016, and December 31, 2018, a total of 141,614 records included the I69 Sequelae of cerebrovascular disease as the main diagnosis or sub-diagnosis. Of these, record form codes indicating dentistry, health center, or psychiatry (n = 1946), and records indicating the type of institution being a psychiatric hospital, dental hospital, postpartum care facility, or health center (n = 99) were excluded. The remaining 139,569 records included the data for inpatient and outpatient care provided at a tertiary hospital/general hospital/hospital, clinic, convalescent hospital, KM hospital, or KM clinic. Among these, records indicating the total cost or number of days in care being 0 or blank were also excluded. Finally, a total of 133,203 records from 19,562 patients were included in the study (Fig. 1).

The numbers of SC and KM patients in the study population were 6,536 and 707 in 2016, 5,903 and 701 in 2017, and 5,527 and 676 in 2018, respectively. This indicated a decreasing trend in both SC and KM treatments over three years (Supplementary Fig. 1, Supplementary Table 1). Patients aged ≥ 75 years accounted for the highest percentage (41.75%) of SC users, and patients aged 65–74 years accounted for the highest percentage (32.58%) of KM users. The percentage of SC and KM users increased from the age of 45 years. The percentage of SC users also increased with increasing age, whereas KM users were relatively evenly distributed among four age groups (45–54, 55–64, 65–74, and ≥ 75 years). Among payer types, the NHI accounted for the highest percentage of payer type for both SC (83.37%) and KM (87.66%) treatments (Table 1).

**Table 1:** Basic characteristics of patients.

Category	Total		Only SC		Only KM		Both SC and KM	
	No. of patients	Percent	No. of patients	Percent	No. of patients	Percent	No. of patients	Percent
**Age**								
< 15	18	0.09	16	0.09	2	0.13	–	–
15–24	50	0.26	48	0.27	1	0.06	1	0.2
25–34	120	0.61	104	0.6	11	0.69	5	1.02
35–44	438	2.24	375	2.15	46	2.88	17	3.48
45–54	1675	8.56	1393	7.97	209	13.1	73	14.96
55–64	3827	19.56	3293	18.84	398	24.94	136	27.87
65–74	5603	28.64	4952	28.33	520	32.58	131	26.84
≥ 75	7831	40.03	7297	41.75	409	25.63	125	25.61
**Gender**								
Male	9494	48.53	8373	47.91	859	53.82	262	53.69
Female	10,068	51.47	9105	52.09	737	46.18	226	46.31
Payer type*								
NHI	16,432	84	14,658	83.87	1399	87.66	375	76.84
Medicaid	3037	15.52	2727	15.6	197	12.34	113	23.16
Others	93	0.48	93	0.53	–	–	–	–

The analysis of the type of visit showed that compared to inpatient care, the percentage of outpatient care was higher among both SC (79.23%) and KM (99.38%) users. Analysis of the type of medical institution revealed that SC users showed the highest percentage of use of hospital level or higher (55.91%) facilities, whereas KM clinic accounted for the highest percentage (89.28%) among KM users (Supplementary Table 2).

### Comparison of the subtypes of KCD-7 code I69

**Figure 2:**
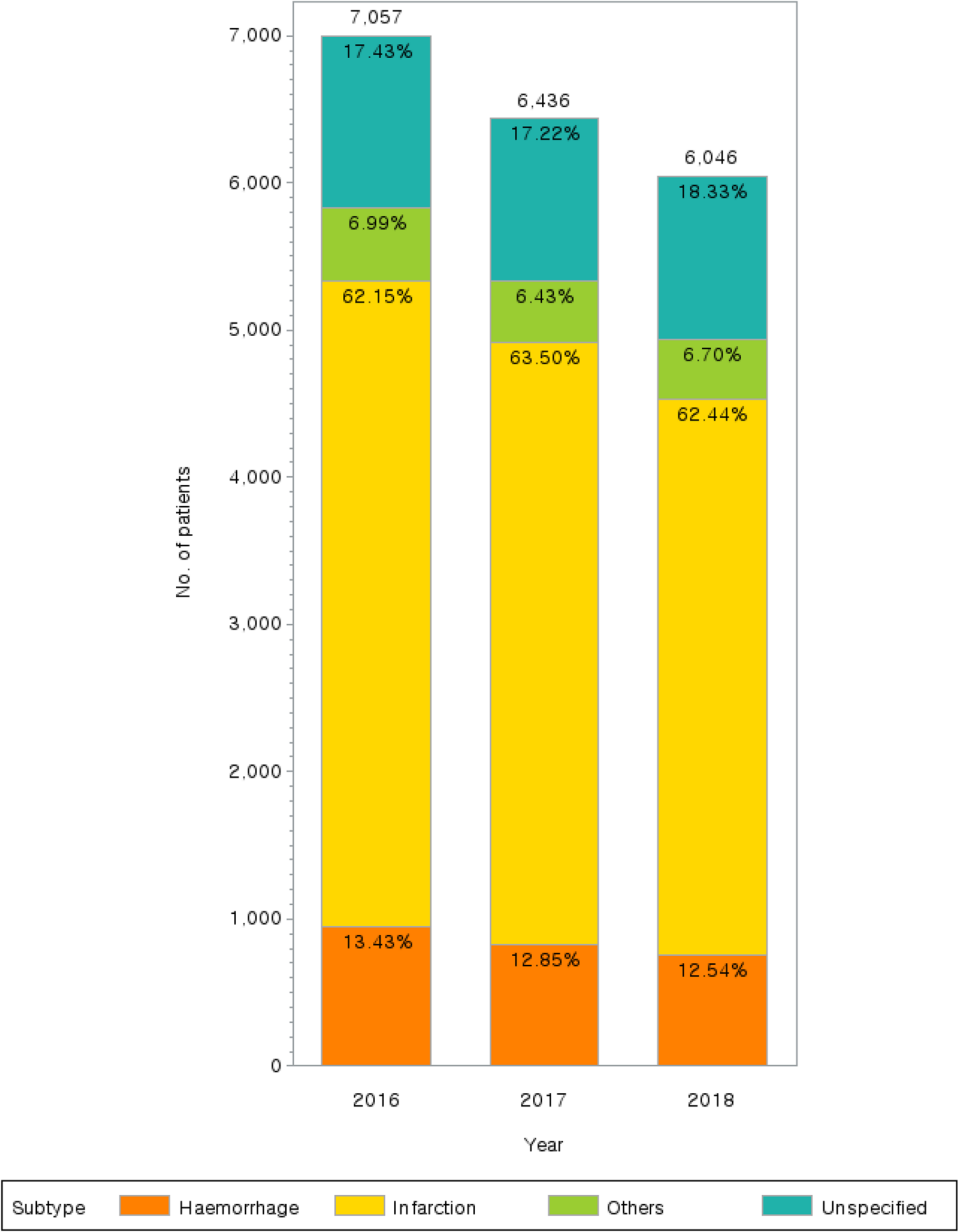
Patients diagnosed with subtypes of code I69. Hemorrhage: Sequelae of subarachnoid hemorrhage (I69.0), Sequelae of intracerebral hemorrhage (I69.1), Sequelae of other nontraumatic intracranial hemorrhage (I69.2); Infarction: Sequelae of cerebral infarction (I69.3); Others: Sequelae of stroke, not specified as hemorrhage or infarction (I69.4); Unspecified: Sequelae of other and unspecified cerebrovascular diseases (I69.8).

The subtypes of diagnosis code I69 were compared by year based on the number of claims (Fig. 2). Infarction accounted for the highest percentage in both SC and KM for all years (62.15% in 2016, 63.50% in 2017, and 62.44% in 2018). The second highest subtype was unspecified, with a 3-year average of 17.66%. However, there were no significant differences in the percentages of subtypes over the three years (p-value = 0.222, Supplementary Table 3).

### Annual rate of change of claims and costs per category of medical service

In SC, the average number of claims showed a decreasing annual trend in terms of the rate of change in all categories. Physical therapy accounted for the highest average annual claims per patient and the highest average annual costs per patient. In KM, the average annual costs per patient showed a distinctly increasing annual trend in rate of change. Injection/procedure fees showed an increase in the average annual claims per patient and the average annual costs per patient (Supplementary Table 4).

### Frequency of health service codes for the sequelae of cerebrovascular disease

Rehabilitation treatment—one of the main treatments in SC—had a 3-year average of 19,158 claims, which was the fourth highest. However, the 3-year average costs per claim ($226) and costs per patient ($2,869.83) for rehabilitation treatment were the second highest after those for hospitalization cost.

Acupuncture—one of the main treatments in KM—showed the highest number of claims, costs per claim, and costs per patient. In particular, acupuncture showed an increasing annual trend in costs per claim. Electroacupuncture stimulation showed an increasing annual trend in the number of claims, increasing from 2974 in 2016 to 3062 in 2017 and 4358 in 2018 (Table 2).

**Table 2:** Frequency of healthcare service codes for sequelae of cerebrovascular disease.

Category		2016			2017			2018		
		Total claims	Annual costs per claims	Annual costs per patient	Total claims	Annual costs per claims	Annual costs per patient	Total claims	Annual costs per claims	Annual costs per patient
SC	Consultation	48,821	$9.18	$43.58	49,072	$9.83	$51.24	43,277	$10.88	$51.07
	Rehabilitation treatment	21,873	$250.14	$2,794.13	21,250	$209.76	$3,137.11	14,351	$218.09	$2,678.26
	Heat therapy	5369	$3.82	$35.13	6085	$3.25	$39.33	4749	$3.02	$30.12
	Injection therapy	5966	$35.83	$65.09	4443	$38.94	$63.08	3437	$34.26	$50.33
	Physical therapy	7183	$89.94	$708.68	6286	$77.96	$817.87	5001	$59.84	$549.94
	Testing	76,296	$45.28	$115.68	62,223	$51.72	$120.86	50,607	$41.25	$95.32
	Treatments	2366	$54.23	$179.90	1195	$87.56	$172.08	1034	$59.27	$134.45
	Hospitalization	32,255	$762.53	$3,914.57	28,440	$1,213.69	$6,140.63	22,862	$1,310.08	$6,255.50
	Prescription/medication	10,441	$10.23	$36.67	11,023	$11.20	$45.88	8939	$10.88	$39.99
KM	Consultation	11,978	$6.84	$104.40	13,556	$7.36	$127.45	11,640	$7.80	$120.75
	Acupuncture	21,075	$7.61	$125.44	22,943	$8.11	$142.81	21,463	$9.33	$147.64
	Moxibustion	4533	$3.04	$45.04	3427	$3.28	$40.33	4374	$4.02	$62.57
	Cupping	4652	$4.44	$63.74	3902	$5.20	$64.03	3819	$5.56	$63.35
	Thermotherapy and cryotherapy of the meridian	6008	$0.85	$12.00	5258	$1.03	$12.01	5693	$1.35	$17.47
	Electroacupuncture stimulation	2974	$4.45	$64.38	3062	$4.56	$64.59	4358	$4.08	$66.64
	Prescription/medication	1717	$0.35	$4.34	2246	$0.43	$5.42	1962	$0.63	$7.48
	Others	203	$108.07	$172.24	195	$118.04	$206.56	180	$150.64	$231.75

*SC* standard care, *KM* Korean medicine.

All costs were converted using the annual average exchange rate (KRW/USD) of the corresponding year. The price level of healthcare cost is adjusted for 2018 (see Supplementary Table 5).

### Prescription and medicine expenses

The most frequently prescribed medicines in the records included cerebrovascular circulation/dementia drugs (Table 3), which were prescribed for a 3-year average of 12,918 cases. The next most frequently prescribed medicines included antithrombotic/anticoagulant agents (n = 11,513), followed by digestants (n = 10,165). With respect to the number of patients who received prescriptions, cerebrovascular circulation/dementia drugs showed the highest 3-year average (3129 patients), followed by antithrombotic/anticoagulant agents and digestants. The number of records and the number of patients showed decreasing trends over the three years. With respect to the annual costs per patient, cerebrovascular circulation/dementia drugs had the highest 3-year average ($182.24). Among records with a 3-year average of 5000 cases or more, all medicines (except vasopressor/blood circulation agents) showed an increasing trend in annual costs per patient over the three years.

**Table 3:** Prescription and medicine costs (Total).

	2016				2017				2018			
Category	No. of claims	No. of patients	Annual costs	Annual costs per patient	No. of claims	No. of patients	Annual costs	Annual costs per patient	No. of claims	No. of Patients	Annual costs	Annual costs per patient
Cerebrovascular circulation/dementia drugs	13,820	3348	$563,261.26	$168.24	13,109	3113	$579,904.81	$186.28	11,825	2926	$562,386.12	$192.20
Antithrombotic/anticoagulant agents	12,679	3105	$372,114.46	$119.84	11,412	2772	$369,553.41	$133.32	10,447	2576	$355,588.46	$138.04
Digestive agents	11,296	3049	$121,319.44	$39.79	10,220	2696	$111,575.01	$41.39	8979	2479	$105,277.27	$42.47
Psychiatric/neurological agents	6,641	1809	$80,606.03	$44.56	5711	1546	$79,644.94	$51.52	5361	1471	$78,477.18	$53.35
Vasopressor/blood circulation agents	6,074	1445	$92,933.43	$64.31	5486	1251	$94,570.96	$75.60	4808	1142	$85,468.79	$74.84
Hyperlipidemia drugs	5,143	1321	$117,078.33	$88.63	5027	1195	$122,707.06	$102.68	4861	1233	$130,833.90	$106.11
Vasodilators	5,647	1454	$75,288.88	$51.78	4,721	1209	$66,182.71	$54.74	4376	1097	$64,679.92	$58.96
Muscle relaxants/NSAIDs	4,387	1376	$34,691.04	$25.21	4268	1244	$34,592.51	$27.81	3352	1075	$28,001.00	$26.05
Anti-diabetic drugs	1,704	408	$12,838.26	$31.47	1405	340	$13,559.35	$39.88	1390	326	$13,739.37	$42.15
Infusion fluids	1,358	767	$14,465.75	$18.86	1,015	628	$11,778.48	$18.76	819	569	$8,428.65	$14.81
Urologicals	1,104	271	$23,871.03	$88.08	1034	238	$22,674.10	$95.27	973	236	$21,484.30	$91.04
Others	2,872	966	$24,729.89	$25.60	2458	856	$20,696.10	$24.18	2038	795	$15,999.62	$20.13

All costs were converted using the annual average exchange rate (KRW/USD) of the corresponding year. The price level of healthcare cost is adjusted for 2018 (see Supplementary Table 5). NSAIDs: nonsteroidal anti-inflammatory drugs.

### Comorbidities

In SC, a total of 1,109 diagnosis codes were identifies as comorbidities. The top 5 most frequent diagnosis codes were essential (primary) hypertension (I10; 8.43%), gastritis and duodenitis (K29; 6.96%), disorders of lipoprotein metabolism and other lipidemia (E78; 6.02%), hemiplegia (G81; 2.98%), dementia in Alzheimer’s disease (F00; 2.73%), and type 2 diabetes mellitus (E11; 2.5%). In total, these 5 diagnosis codes accounted for 27.12% of all diagnosis codes. With the inclusion of cerebral infarction (I63), other functional intestinal disorders (K59), disorders of vestibular function (H81), gastro-esophageal reflux disease (K21), depressive episode (F32), dizziness and giddiness (R42), other anxiety disorders (F41), and dorsalgia (M54), these codes accounted for 45.05% of all diagnosis codes. When categorized according to the KCD-7 code, the top 5 most frequent categories were diseases of the circulatory system (I00–I99; 18.0%); diseases of the digestive system (K00–K93; 15.1%); endocrine, nutritional, and metabolic diseases (E00–E90; 11.6%); mental and behavioral disorders (F00–F99; 11.2%); and diseases of the nervous system (G00–G99; 10.3%). Collectively, these 5 categories accounted for 66.2% of all categories.

In KM, a total of 404 diagnosis codes were identified as comorbidities. The top 5 most frequent diagnosis codes were dorsalgia (M54; 7.15%), other soft tissue disorders NEC (M79; 5.86%), hemiplegia (G81; 2.98%), essential (primary) hypertension (I10; 4.58%), and gastritis and duodenitis (K29; 3.71%). In total, these 5 diagnosis codes accounted for 26.26% of all diagnosis codes. With the inclusion of disorders of lipoprotein metabolism and other lipidemia (E78), other joint disorders NEC (M25), functional dyspepsia (K30), other disorders of muscle (M62), gonarthrosis [arthrosis of knee] (M17), type 2 diabetes mellitus (E11), other functional intestinal disorders (K59), shoulder lesions (M75), and dizziness and giddiness (R42), these codes accounted for 46.71% of all diagnosis codes. When categorized according to the KCD-7 code, the top 5 most frequent categories were diseases of the musculoskeletal system and connective tissue (M00–M99; 28.1%); diseases of the digestive system (K00–K93; 11.7%); symptoms, signs and abnormal clinical and laboratory findings NEC (R00–R99, 10.8%); diseases of the nervous system (G00–G99; 10.7%); and diseases of the circulatory system (I00–I99; 10.2%). Collectively, these 5 categories accounted for 71.5% of all categories.

Essential (primary) hypertension (I10), gastritis and duodenitis (K29), and disorders of lipoprotein metabolism and other lipidemia (E78) accounted for approximately 21.41% of all comorbidities in SC, and for a relatively lower percentage (11.89%) in KM. In contrast, dorsalgia (M54) and other soft tissue disorders NEC (M79) accounted for approximately 13.01% of all comorbidities in KM, and for a relatively lower percentage (~ 2.38%) in SC (Table 4).

**Table 4:** Comorbidities of patients with different diagnosis codes.

Standard care				Korean medicine			
Code	Diagnosis name	No. of patients	Percent (%)	Code	Diagnosis name	No. of patients	Percent (%)
I10	Essential(primary) hypertension	8272	8.43	M54	Dorsalgia	429	7.15
K29	Gastritis and duodenitis	6832	6.96	M79	Other soft tissue disorders, NEC	352	5.86
E78	Disorders of lipoprotein metabolism and other lipidemia	5910	6.02	G81	Hemiplegia	298	4.96
G81	Hemiplegia	2923	2.98	I10	Essential(primary) hypertension	275	4.58
F00	Dementia in Alzheimer’s disease (G30.- +)	2683	2.73	K29	Gastritis and duodenitis	223	3.71
E11	Type 2 diabetes mellitus	2683	2.73	E78	Disorders of lipoprotein metabolism and other lipidemia	216	3.6
I63	Cerebral infarction	2235	2.28	M25	Other joint disorders, NEC	171	2.85
K59	Other functional intestinal disorders	2021	2.06	K30	Functional dyspepsia	160	2.66
H81	Disorders of vestibular function	1988	2.03	M62	Other disorders of muscle	147	2.45
K21	Gastro-esophageal reflux disease	1909	1.95	M17	Gonarthrosis[arthrosis of knee]	119	1.98
F32	Depressive episode	1836	1.87	E11	Type 2 diabetes mellitus	110	1.83
R42	Dizziness and giddiness	1753	1.79	K59	Other functional intestinal disorders	107	1.78
F41	Other anxiety disorders	1718	1.75	M75	Shoulder lesions	100	1.67
M54	Dorsalgia	1446	1.47	R42	Dizziness and giddiness	98	1.63
F01	Arteriosclerotic dementia	1370	1.4	I63	Cerebral infarction	90	1.5
F06	Other mental disorders due to brain damage and dysfunction and to physical disease	1098	1.12	J00	Acute nasopharyngitis	81	1.35
R51	Headache	962	0.98	R51	Headache	79	1.32
G40	Epilepsy	927	0.94	F32	Depressive episode	72	1.2
M79	Other soft tissue disorders, NEC	895	0.91	K21	Gastro-esophageal reflux disease	66	1.1
K30	Functional dyspepsia	876	0.89	H81	Disorders of vestibular function	62	1.03

## Discussion

The present study used HIRA-NPS data (2016–2018) to identify the demographic characteristics and diagnosis code subtypes of patients diagnosed with I69 Sequelae of cerebrovascular disease, and to analyze the medical service utilization of SC and KM users from a healthcare third-party payer perspective. Among these patients, 84.45% received outpatient care. Moreover, 55.91% of SC users visited facilities classified as hospital level or above, whereas 89.28% of KM users visited KM clinics. During the three years examined in the study, the number of claims and healthcare costs decreased in SC, with physical therapy and rehabilitation treatment accounting for the highest percentages. However, during the same period, healthcare costs increased in KM, with injection/procedure fees and acupuncture accounting for the highest percentages. With respect to comorbidities, we identified several diagnosis codes associated with risk factors of stroke in SC, and with musculoskeletal and connective tissue disorders in KM.

Diagnosis code I69 includes chronic post-stroke sequelae, as well as various other symptoms that appear in stroke survivors^4,5^. The number of patients with this diagnosis code decreased steadily among SC and KM users over the three years. These findings are inconsistent with the results of studies in the US and other countries. Previous studies have reported that the prevalence of stroke is increasing, whereas the mortality rate of strokes is decreasing^4^, implying that the number of patients with post-stroke sequelae is increasing^5,6^. In South Korea, the mortality rate of stroke decreased from 58.9 per 100,000 population in 2006 to 29.6 per 100,000 population in 2015. However, the results of a previous study did not reveal any specific trends in the prevalence of stroke among patients aged ≥ 50 years during 1998–2014^46^. Previous studies have attributed this lack of any trend to limitations of the data, and have attempted to overcome these limitations with additional data^46^. On the other hand, it is noteworthy to mention that only the patients with conclusive neurological and radiological evidence of post-stroke sequelae, instead of other lasting symptoms due to comorbidities, would have been included in the national health insurance claims data, which would have played a role in the decreasing trend^47^. Considering the findings in the present study, we suggest that additional studies are needed to elucidate these discrepancies in observed and expected trends.

Among both SC and KM users, infarction occurred most frequently in all three years. Ischemic stroke—which includes cerebral infarction—accounts for the highest percentage of all stroke events, and has a higher survival rate than other cerebrovascular diseases. For this reason, it is believed to show a high prevalence among patients with sequelae^48^. It is noteworthy that in Korea, premature discontinuation of antiplatelets after ischemic stroke was higher in the rural areas than in the urban areas^49^. Considering this issue, future studies are required to examine the differences in the healthcare utilization of KM and SC between urban and rural areas of Korea among patients suffering from post-stroke sequelae.

Medical institutions classified at a hospital level or higher were the highest utilized facilities among SC users, whereas KM patients primarily utilized KM clinics. The higher utilization of hospitals or higher-level facilities in SC may be related to rehabilitation treatment. With respect to the number of claims by service category and high-frequency service codes, rehabilitation treatment was the main treatment modality in SC for post-stroke sequelae. For post-stroke rehabilitation treatment, the rehabilitation treatment teams are multidisciplinary teams comprising rehabilitation medicine specialists, rehabilitation nurses, physical therapists, and occupational therapists^50,51^. This is also recommended by domestic treatment guidelines^52^. Therefore, it would be easier to operate rehabilitation treatment programs at hospitals or facilities classified at a higher level than clinics, which may explain our observed results.

In KM treatment, KM clinics were utilized to a higher degree than KM hospitals. This may be associated with the accessibility of medical institutions for acupuncture and comorbidities. Acupuncture was the main treatment modality, and musculoskeletal pain and disorders were the major comorbidities in KM. A 2017 survey of KM utilization reported that there were more KM clinics (n = 13,868) than KM hospitals (n = 282) as of 2016^53^. Accordingly, the utilization of KM clinics may have been higher among KM users because they provide better access to acupuncture treatment for post-stroke musculoskeletal pain and disorders.

The average annual number of claims decreased for all categories in SC. Moreover, the average annual claims per patient also decreased in most categories, including testing and diagnostic radiology and radiotherapy. It could be attributed to the decrease in the total number of patients and claims in SC. In KM, the average annual number of claims did not decrease significantly. It could be because the decreasing trends in the total number of patients and claims are less severe in KM. We found a relative decrease in the annual rate of change in hospitalization fees. However, it does not seem to be meaningful results due to the absolute number of cases being too low. Moreover, the service categories in KM did not include physical therapy, treatment and surgery, anesthesia, diagnostic radiology and radiotherapy, special equipment fee, and psychotherapy. Although South Korea has a dichotomized healthcare system for both SC and KM^13,14^, the aforementioned treatment categories are not covered by insurance for KM. Therefore, such information was not included in the data used in the study.

Health expenditure showed different trends by categories and types of medicines. For SC, the total expense (Supplementary Fig. 1C) as well as the annual expense per patient (Supplementary Fig. 1D) decreased; however, the annual change rate in the cost per patient (Supplementary Table 4) shows that decreasing trend is prominent in treatment procedure categories such as injection, physical therapy, and surgery, while diagnostic, consulting, and hospitalization fees showed mild to moderate increase. On the other hand, all categories in KM claims except for testing showed an increasing trend. Although direct cause of the different trend is difficult to identify from our database, this decrease of overall cost may be partially attributable to difficulties in confirmatory diagnosis of post-stroke sequelae, which may explain why diagnostic fees increased and treating fees decreased^47^. In addition, the prognosis of stroke patients in Korea may have improved in general due to the decrease in onset-to-treatment time and increased thrombolysis usage^54^, leading to a reduced healthcare utilization due to post-stroke sequelae. The total cost and annual expense per patient in KM remained constant or mildly increasing, although the absolute value was very low. This may be partially explained by the patients’ preference towards KM treatment in the chronic symptoms from stroke attack^55^, and the development of clinical practice guidelines for stroke rehabilitation^56^, but further studies are necessary to clearly identify the underlying factors of different trends.

In SC, physical therapy included rehabilitation treatment (including rehabilitative development therapy for disorders of the central nervous system, rehabilitative functional training, activities of daily living training, exercise therapy, and occupational therapy), heat therapy (superficial/deep heat therapy), and physical therapy (including TENS, interferential current therapy, and functional electrical stimulation therapy). Physical therapy was prescribed in 12.73% of all cases. Moreover, the average annual costs per patient accounted for 21.50% of all costs, indicating higher costs relative to the number of claims. Excluding hospitalization-related costs, rehabilitation treatment had the highest annual costs per claim and highest annual costs per patient in all years, but accounted for the fourth highest number of claims. Rehabilitation treatment is strongly recommended in SC for treating post-stroke sequelae. Previous studies have reported that post-stroke rehabilitation treatment is effective in improving activities of daily living and in reducing disability^52^. Our findings also show that rehabilitation treatment is widely used in clinical practice due to its excellent effects.

In KM, injection/procedure fees had the highest cost and accounted for a high percentage of the number of claims. However, the average annual costs per patient (12.30%) were low relative to the number of claims (69.83%). We believe that the high percentage of injection/procedure fees is due to acupuncture being included in this category. Due to the high frequency of prescriptions, acupuncture accounted for most of the claim cases, had the highest annual costs per patient, and also showed an increasing trend in annual costs per claim. Acupuncture is the most commonly used medical service in KM^53^, and patients with sequelae of stroke (diagnosis code I69) also use acupuncture with the highest frequency. Previous study shows that acupuncture treatment in mild to moderate ischemic stroke reduce mortality risk and shows a tendency of reduced recurrence than in non-treated patients^57^.

Previous studies have already reported the efficacy of acupuncture for post-stroke sequelae^17–19,58–60^. Acupuncture treatment during the post-stroke period has beneficial effects on improving activities of daily living and neurological impairments, including motor and cognitive functions. Moreover, it can also be beneficial for musculoskeletal pain and impairment and for the rehabilitation of psychiatric sequelae, such as depression and anxiety^17^. Furthermore, electroacupuncture stimulation showed an increasing trend in the number of claims in KM. Previous studies have demonstrated that among patients with post-stroke sequelae, electroacupuncture stimulation is effective for several musculoskeletal disorders, including spasticity, motor function problems, and reflex sympathetic dystrophy^20–22^. In the present study, the major comorbidities in KM were found to include dorsalgia (M54), other soft tissue disorders (M79), hemiplegia (G81), other joint disorders NEC (M25), other disorders of muscle (M62), and gonarthrosis [arthrosis of knee] (M17). Based on disease classification codes, diseases of the musculoskeletal system and connective tissue (M00–M99) accounted for close to 30% of the comorbidities. The results suggest that acupuncture and electroacupuncture stimulation are widely used as treatment modalities in KM clinical practice for post-stroke sequelae and associated musculoskeletal pain, with an emphasis on symptom improvement.

The records showed that cerebrovascular circulation/dementia drugs were prescribed most often. The recommended treatment of post-stroke dementia includes controlling vascular risk factors with additional pharmacological treatment through a symptomatic approach^61^. However, in South Korea, the medicines for cognitive impairment—such as choline alfoscerate and L-carnitine—are covered by insurance through the NHIS. The over-prescription of these drugs has led to the deterioration of health insurance finances, and poses health risks to patients taking multiple drugs^62,63^. As a result, the cost burden on patients appears to have increased. Additional studies are needed on the clinical efficacy and economic feasibility of drugs that were not investigated in the present study.

Antithrombotic/anticoagulant agents were prescribed at the second highest frequency, and are generally used for the treatment of ischemic stroke and for secondary prevention of intracerebral hemorrhage^64,65^. In addition, {Viswanathan, 2006 #94} vasopressor/blood circulation agents are used to regulate hypertension, which is a risk factor of stroke^66^. Thus, the use of the drugs may be associated with the prevention stroke recurrence. The major comorbidities in KM—essential (primary) hypertension (I10), disorders of lipoprotein metabolism and other lipidemia (E78), and type 2 diabetes mellitus (E11)—were consistent with the risk factors for stroke^4,41,46^. These results indicate that patients with diagnosis code I69 were still exposed to the risk of stroke recurrence simultaneous with post-stroke sequelae. Therefore, there is a need for continued pharmacological treatment and management for such risk factors. Digestive agents were the third most frequently prescribed medicine, and included digestive enzymes and drugs for gastrointestinal motility, constipation, and heartburn. The use of these drugs may be associated with the high percentages of gastritis and duodenitis (K29) and gastro-esophageal reflux disease (K21) among the comorbidities. On the other hand, although phytotherapeutics and herbal medicines are not covered by the National Health Insurance in Korea and therefore could not be analyzed in this study, previous study shows that Chinese herbal medicine can be used and is effective in post-stroke functional recovery^67^. Further studies including clinical trials will help understand the effect of other types of phytotherapeutics in post-stroke sequelae.

### Study limitations and strengths

The present study has some limitations. First, there may be inconsistencies between the claims records and the actual diseases. In the NHIS system, claims records and diagnosis codes are utilized for the reimbursement of medical service providers. Therefore, medical service providers may have an incentive to pursue a bigger reimbursement rather than list the disease accurately^68^. Moreover, the diagnosis codes investigated in the present study (I69 Sequelae of cerebrovascular disease) are inputted after the causes of sequelae (I60–I67.1 and I67.4–I67.9) have already been inputted. In such cases, the medical institution may omit entries during this process. Consequently, the claims data may not accurately reflect the disease. This is an inherent problem of data accumulation that should be considered when using such data. Second, the present study used the HIRA-NPS as the data source. However, these data are non-continuous annual claims data that do not include clinical test values. Therefore, it is difficult to identify the severity of the disease and the stroke-related comorbidities from such data^68^. The subtypes of I69 (I69.0–I69.4 and I69.8) also do not provide information about the severity of the disease. All HIRA data are assigned specific symbols (codes) by the government based on administrative rules, and these codes are used to extend health insurance for patients with severe diseases. However, this information is not enough to estimate the severity of disease. Third, the study population included only patients who received medical services covered by insurance. Therefore, our results do not reflect the utilization of services not covered by insurance. In KM, a total 201 services covered by insurance were included in the claims data; however, this is far lower than the 5611 services covered in SC^69^. The Korean Medicine Clinical Practice Guidelines for Stroke^15^ recommend the use of herbal medicine for the treatment of post-stroke sequelae, and herbal medicines are used in clinical practice as well. However, these are not covered by insurance, and were not included in the present study.

Nonetheless, the present study is significant in that it is the first study to analyze medical service utilization among patients with post-stroke sequelae within the bounds of the dichotomized healthcare system in South Korea^13,14^. According to validation studies, the diagnostic accuracy of cerebrovascular diseases in the NHIS is greater than 80 percent^70–72^. Moreover, the databased used in this study (HIRA-NPS) is representative of the large-scale population demographics of South Korea, with sufficient representativeness across all age groups and regions^45^. Therefore we were able to conclude that our findings reflect the trends in medical service utilization from post-stroke sequelae in Korea during the three most recent years.

Although the study subjects showed similarities in some demographic characteristics and diagnosis subtypes, they showed differences in the frequency and trends in cost of care for medical service categories and comorbidities. The findings in the present study can be used for additional studies on SC and KM practices related to post-stroke sequelae. Moreover, these findings could also be used as supporting data for reasonable decision making by clinicians who provide care to patients with post-stroke sequelae, and may be utilized as basic data in integrative SC/KM studies. Furthermore, our findings should also be used to inform public health policy decisions regarding budget and coverage items.

## Conclusion

We compared and analyzed the medical service utilization and characteristics of patients diagnosed with I69 Sequelae of cerebrovascular disease separately for SC and KM using the 2016–2018 HIRA-NPS data. Although SC and KM users showed similarities in some demographic characteristics and diagnosis subtypes, they showed differences in the number of medical service claims, cost of care, and comorbidities. Additional studies are needed to address the limitations of the sample data.

## Supplementary Information

Supplementary Information.

## Data Availability

The Health Insurance Service & Assessment Service in Korea provides the HIRA-NPS. Due to privacy and ethical concerns, the datasets utilized or analyzed in this study are not publicly available. (https://opendata.hira.or.kr/op/opc/selectPatDataAplInfoView.do).

## References

[1] Teasell RW (1992). Long-term sequelae of stroke: How should you handle stroke complications?. Can. Fam. Phys. Med. Fam. Canadien.

[2] Centers for Disease Control and Prevention (2009). Prevalence and most common causes of disability among adults–United States, 2005. MMWR. Morb. Mortal. Wkly Rep.

[3] Coull AJ, Lovett JK, Rothwell PM (2004). Population based study of early risk of stroke after transient ischaemic attack or minor stroke: Implications for public education and organisation of services. BMJ (Clinical Research ed.).

[4] Benjamin EJ (2019). Heart disease and stroke statistics-2019 update: A report from the American heart association. Circulation.

[5] Chang CC (2016). Characteristics of traditional Chinese medicine usage in patients with stroke in Taiwan: A nationwide population-based study. J. Ethnopharmacol..

[6] Feigin VL (2014). Global and regional burden of stroke during 1990–2010: Findings from the global burden of disease study 2010. Lancet.

[7] Westerlind E, Singh R, Persson HC, Sunnerhagen KS (2020). Experienced pain after stroke: A cross-sectional 5-year follow-up study. BMC Neurol..

[8] Dorňák T (2019). Prevalence and evolution of spasticity in patients suffering from first-ever stroke with carotid origin: A prospective, longitudinal study. Eur. J. Neurol..

[9] Klit H, Finnerup NB, Jensen TS (2009). Central post-stroke pain: Clinical characteristics, pathophysiology, and management. Lancet Neurol..

[10] Lendraitienė E, Tamošauskaitė A, Petruševičienė D, Savickas R (2017). Balance evaluation techniques and physical therapy in post-stroke patients: A literature review. Neurol. Neurochir. Pol..

[11] Ferro JM, Caeiro L, Figueira ML (2016). Neuropsychiatric sequelae of stroke. Nat. Rev. Neurol..

[12] Jones CA, Colletti CM, Ding M-C (2020). Post-stroke dysphagia: Recent insights and unanswered questions. Curr. Neurol. Neurosci. Rep..

[13] Bodeker G (2001). Lessons on integration from the developing world’s experience traditional medicine. BMJ (Clinical research ed.).

[14] Jung, B., Bae, S. & Kim, S. Use of Western medicine and traditional Korean medicine for joint disorders: A retrospective comparative analysis based on Korean nationwide insurance data. *Evid.-Based Complement. Altern. Med.* **2017** (2017). 10.1155/2017/2038095 PMC5804363 29456569

[15] The society for stroke on Korean medicine. Korean medicine clinical practice guideline for stroke. 1–454 (2021).

[16] Venketasubramanian N (2021). Complementary and alternative interventions for stroke recovery - A narrative overview of the published evidence. J. Complement. Integr. Med..

[17] Yang, A. *et al.* Acupuncture for stroke rehabilitation. *Cochrane Database Syst. Rev.* (2016). 10.1002/14651858.CD004131.pub3 PMC6464684 27562656

[18] Lee JA (2012). Acupuncture for shoulder pain after stroke: A systematic review. J. Altern. Complement. Med..

[19] Fan W (2020). Acupuncture therapy for poststroke spastic hemiplegia: a systematic review and meta-analysis of randomized controlled trials. Complement. Ther. Clin. Pract..

[20] Zhan J (2018). Electroacupuncture as an adjunctive therapy for motor dysfunction in acute stroke survivors: A systematic review and meta-analyses. BMJ Open.

[21] Cai Y (2017). Electroacupuncture for poststroke spasticity: A systematic review and meta-analysis. Arch. Phys. Med. Rehabil..

[22] Wei X (2019). Electroacupuncture for reflex sympathetic dystrophy after stroke: A meta-analysis. J. Stroke Cerebrovasc. Dis..

[23] Li X (2021). Moxibustion for post-stroke urinary incontinence in adults: A systematic review and meta-analysis of randomized controlled trials. Complement. Ther. Clin. Pract..

[24] Wen J (2018). Treatment of poststroke constipation with moxibustion: A case report. Medicine.

[25] Wei YX, Zhao X, Zhang BC (2016). Synergistic effect of moxibustion and rehabilitation training in functional recovery of post-stroke spastic hemiplegia. Complement. Ther. Med..

[26] Han SY, Hong ZY, Xie YH, Zhao Y, Xu X (2017). Therapeutic effect of Chinese herbal medicines for post stroke recovery: A traditional and network meta-analysis. Medicine.

[27] Zhang H, Li M, Xu T (2021). Therapeutic effect of Chinese herbal medicines for post-stroke depression: A meta-analysis of randomized controlled trials. Medicine.

[28] Xu L (2020). Adjuvant therapy with Astragalus membranaceus for post-stroke fatigue: A systematic review. Metab. Brain Dis..

[29] Cai Y (2019). Add-on effects of chinese herbal medicine for post-stroke spasticity: A systematic review and meta-analysis. Front. Pharmacol..

[30] Kwon CY, Lee B, Chung SY, Kim JW (2019). Herbal medicine for post-stroke anxiety: A systematic review and meta-analysis of randomized controlled trials. Complement. Ther. Clin. Pract..

[31] Kwon S (2018). Health-related quality of life and related factors in stroke survivors: Data from Korea National Health and Nutrition Examination Survey (KNHANES) 2008 to 2014. PLoS ONE.

[32] Lee Y, Kim WS, Paik NJ (2017). Gender differences in physical activity and health-related behaviors among stroke survivors: Data from the 5th Korea National Health and Nutrition Examination Survey. Top. Stroke Rehabili..

[33] Kim M, Oh GJ, Lee YH (2017). Association between stroke status and depression in a community setting: The 2014 Korea National Health and Nutrition Examination Survey. J. Clin. Neurol. (Seoul, Korea).

[34] Yoon JA (2017). Factors associated with improvement or decline in cognitive function after an ischemic stroke in Korea: The Korean stroke cohort for functioning and rehabilitation (KOSCO) study. BMC Neurol..

[35] Shin YI (2018). Association between spasticity and functional impairments during the first year after stroke in Korea: The KOSCO study. Am. J. Phys. Med. Rehabil..

[36] Yu KH (2013). Cognitive impairment evaluated with Vascular Cognitive Impairment Harmonization Standards in a multicenter prospective stroke cohort in Korea. Stroke.

[37] Kim BJ (2015). Case characteristics, hyperacute treatment, and outcome information from the clinical research center for stroke-fifth division registry in South Korea. J. Stroke.

[38] Jeong HY (2020). Characteristics and management of stroke in Korea: 2014–2018 data from Korean stroke registry. Int. J. Stroke Off. J. Int. Stroke Soc..

[39] Hong KS (2011). Burden of ischemic stroke in Korea: Analysis of disability-adjusted life years lost. J. Clin. Neurol. (Seoul, Korea).

[40] Hong KS (2013). Stroke statistics in Korea: Part II stroke awareness and acute stroke care, a report from the Korean stroke society and clinical research center for stroke. J. Stroke.

[41] Hong K-S (2013). Stroke statistics in Korea: Part I. Epidemiology and risk factors: A report from the korean stroke society and clinical research center for stroke. J. Stroke.

[42] Kim M, Yun SM, Jeong J, Jo C, Koh YH (2020). Association between blood lead level and risk of stroke in Korean adults: A cross-sectional study in the Korea National Health and Nutrition Examination Survey 2008–2013. BMJ Open.

[43] Seo YG, Choi HC, Cho B (2016). The relationship between metabolically obese non-obese weight and stroke: The Korea National Health and Nutrition Examination Survey. PLoS ONE.

[44] Lee WJ (2018). Relationship between open-angle glaucoma and stroke: A 2010 to 2012 Korea National Health and Nutrition Examination Survey. J. Glaucoma.

[45] Kim L, Kim JA, Kim S (2014). A guide for the utilization of Health Insurance Review and Assessment Service National Patient Samples. Epidemiol. Health.

[46] Kim JY (2019). Executive summary of stroke statistics in Korea 2018: A report from the Epidemiology Research Council of the Korean Stroke Society. J. Stroke.

[47] Ikeda S (2021). A nationwide multi-center questionnaire survey on the real-world state and issues regarding post-stroke complications in Japan. J. Stroke Cerebrovasc. Dis..

[48] Jun Yup, K. *et al.* Executive Summary of Stroke Statistics in Korea 2018: A Report from the Epidemiology Research Council of the Korean Stroke Society. *J. stroke* **21**, 42–59. 10.5853/jos.2018.03125 (2019). 10.5853/jos.2018.03125 PMC6372894 30558400

[49] Kim SJ (2021). Prevalence and associated factors of premature discontinuation of antiplatelet therapy after ischemic stroke: A nationwide population-based study. BMC Neurol.

[50] Langhorne P, Pollock A (2002). What are the components of effective stroke unit care?. Age Ageing.

[51] Dobkin BH (2003). The Clinical Science of Neurologic Rehabilitation.

[52] Kim DY (2017). Clinical practice guideline for stroke rehabilitation in Korea 2016. Brain Neurorehabil..

[53] Ministry of Health and Welfare, National Development Institute of Korean Medicine. Report on Usage and Consumption of Korean Medicine 2017; Basic report (for citizens). https://www.koms.or.kr/board/researchReport/view.do?post_no=45&menu_no=21. Accessed 28 Nov 2022. [In Korean]

[54] Kim H (2021). Golden hour thrombolysis in acute ischemic stroke: The changing pattern in South Korea. J. Stroke.

[55] Park M, Hunter J, Kwon S (2018). Evaluating integrative medicine acute stroke inpatient care in South Korea. Health Policy.

[56] Kim, D. Y. *et al.* Clinical practice guideline for stroke rehabilitation in Korea 2016. *Brain Neurorehabil.* **10** (2017). 10.12786/bn.2023.16.e18 PMC10404807 37554256

[57] Lee Y-S, Kwon S, Chae Y, Jang B-H, Ko S-G (2018). A retrospective cohort study on the outcomes of ischemic stroke patients with adjuvant Korean Medicine treatment. Sci. Rep..

[58] Amorim D (2018). Acupuncture and electroacupuncture for anxiety disorders: A systematic review of the clinical research. Complement. Ther. Clin. Pract..

[59] Long Y-B, Wu X-P (2012). A meta-analysis of the efficacy of acupuncture in treating dysphagia in patients with a stroke. Acupunct. Med..

[60] Liu F, Li Z-M, Jiang Y-J, Chen L-D (2014). A meta-analysis of acupuncture use in the treatment of cognitive impairment after stroke. J. Altern. Complement. Med..

[61] Leys D, Hénon H, Mackowiak-Cordoliani M-A, Pasquier F (2005). Poststroke dementia. Lancet Neurol..

[62] Moon Y, Lim J-S, Lee C-N, Choi H (2020). Vulnerable strata to non-adherence and overuse in treatment for patients with cognitive impairment. Dement. Neurocognitive Disord..

[63] Eun-young, K. *Korea Biomedical Revies* (Yang Kyung-cheol, 76–1, Dongmak-ro, Mapo-gu, Seoul, Korea, 2021).

[64] Viswanathan A (2006). Antiplatelet use after intracerebral hemorrhage. Neurology.

[65] Yaghi S (2020). Anticoagulation type and early recurrence in cardioembolic stroke: The IAC study. Stroke.

[66] Hankey GJ (2017). Stroke. Lancet (London, England).

[67] Tseng CY (2022). Acupuncture and traditional Chinese herbal medicine integrated with conventional rehabilitation for post-stroke functional recovery: A retrospective cohort study. Front. Neurosci..

[68] Kim J-A, Yoon S, Kim L-Y, Kim D-S (2017). Towards actualizing the value potential of Korea Health Insurance Review and Assessment (HIRA) data as a resource for health research: Strengths, limitations, applications, and strategies for optimal use of HIRA data. J. Korean Med. Sci..

[69] Ryu H-S (2020). Analysis of medical services provided to patients with ankle sprains in Korea between 2015 and 2017: a cross-sectional study of the health insurance review and assessment service national patient sample database. BMJ Open.

[70] Kim JY (2020). Development of stroke identification algorithm for claims data using the multicenter stroke registry database. PLoS ONE.

[71] Park TH, Choi JC (2016). Validation of stroke and thrombolytic therapy in Korean National Health Insurance claim data. J. Clin. Neurol..

[72] Park J-K (2000). The accuracy of ICD codes for cerebrovascular diseases in medical insurance claims. J. Prev. Med. Public Health.

