# Supplementary material for: Analysis of medical service utilization for post-stroke sequelae in Korea between 2016 and 2018: a cross-sectional study
Source: Sci Rep. 2022 Nov 28;12:20501. doi: 10.1038/s41598-022-24710-8 (PMC9705313; doi:10.1038/s41598-022-24710-8)
Supplement: Supplementary file 1 — Supplementary Information. [file 41598_2022_24710_MOESM1_ESM.docx]

**Title**

Analysis of medical service utilization for post-stroke sequelae in Korea between 2016 and 2018: a cross-sectional study

**Authors**

Hyun-Jun Lee, Yu-Cheol Lim, Ye-Seul Lee, In-Hyuk Ha

**Supplementary Figure 1. General medical services utilization among patients with post-stroke sequelae in Korea.** Patients can be counted in more than one category. All costs were converted using the annual average exchange rate (KRW/USD) for the corresponding year. The healthcare costs were adjusted according to the health sector consumer price index for 2018 (see Supplementary Table 5). SC: Standard care; KM: Korean Medicine

**
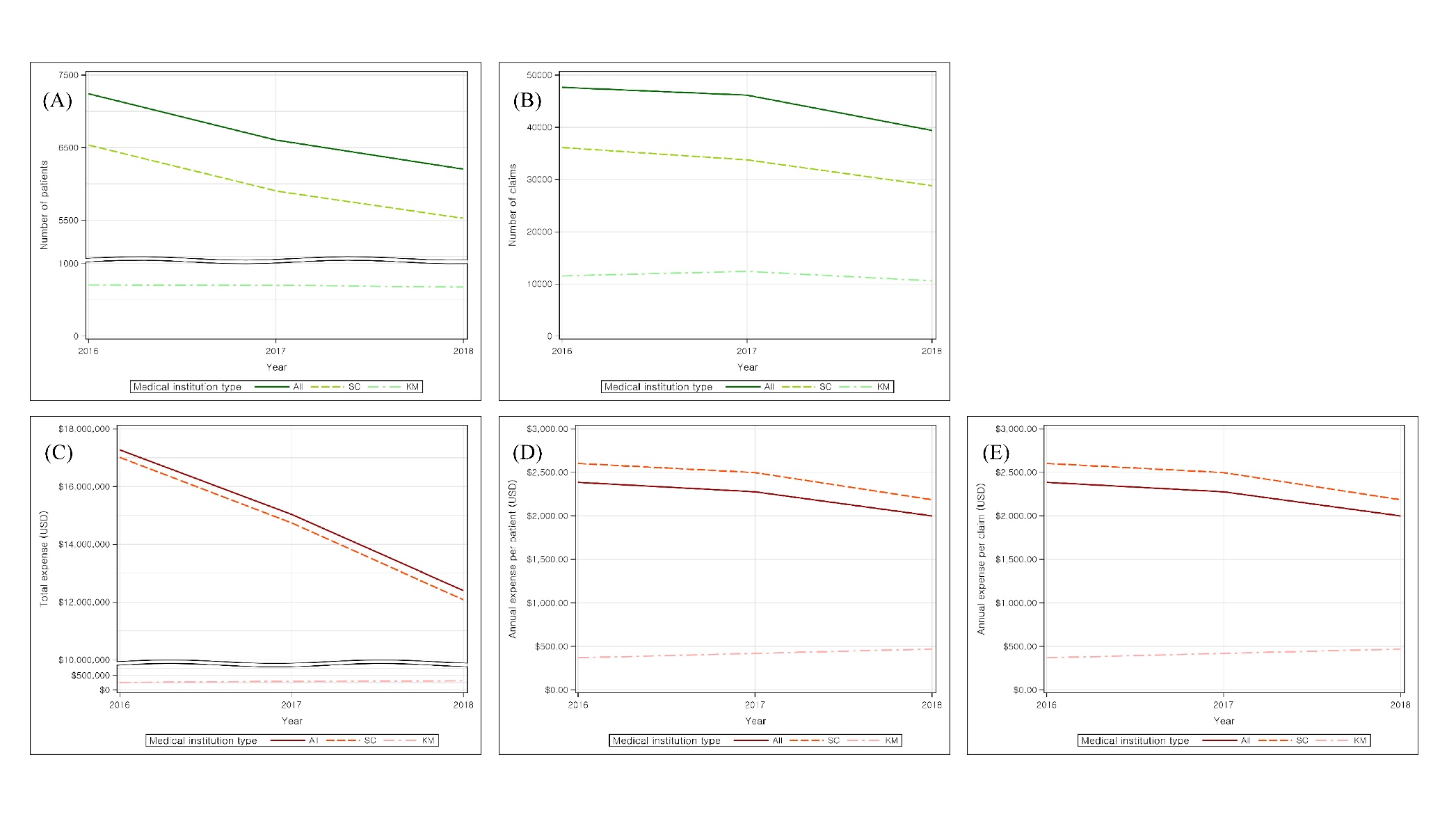
**

**Supplementary Table 1. General medical services utilization among patients with post-stroke sequelae in Korea.** Patients can be counted in more than one category. All costs were converted using the annual average exchange rate (KRW/USD) for the corresponding year. The healthcare costs were adjusted according to the health sector consumer price index for 2018 (see Supplementary Table 5).

| \| **Year** \| **Types** \| **No. of patients*** \| **No. of claims** \| **Annual costs** \| **Annual costs per patient** \| **Annual costs per claim** \| \| --- \| --- \| --- \| --- \| --- \| --- \| --- \| \| 2016 \| Total \| 7243 \| 47653 \| $17,274,637.14 \| $2,385.01 \| $362.51 \| \| Standard Care \| 6536 \| 36112 \| $17,013,502.29 \| $2,603.05 \| $471.13 \| \| Korean Medicine \| 707 \| 11541 \| $261,134.85 \| $369.36 \| $22.63 \| \| 2017 \| Total \| 6604 \| 46156 \| $15,035,171.54 \| $2,276.68 \| $325.75 \| \| Standard Care \| 5903 \| 33754 \| $14,740,862.23 \| $2,497.18 \| $436.71 \| \| Korean Medicine \| 701 \| 12402 \| $294,309.31 \| $419.84 \| $23.73 \| \| 2018 \| Total \| 6203 \| 39394 \| $12,397,388.10 \| $1,998.61 \| $314.70 \| \| Standard Care \| 5527 \| 28804 \| $12,079,633.80 \| $2,185.57 \| $419.37 \| \| Korean Medicine \| 676 \| 10590 \| $317,754.30 \| $470.05 \| $30.01 \| |
| --- | --- | --- | --- | --- | --- | --- | --- | --- | --- | --- | --- | --- | --- | --- | --- | --- | --- | --- | --- | --- | --- | --- | --- | --- | --- | --- | --- | --- | --- | --- | --- | --- | --- | --- | --- | --- | --- | --- | --- | --- | --- | --- | --- | --- | --- | --- | --- | --- | --- | --- | --- | --- | --- | --- | --- | --- | --- | --- | --- | --- | --- | --- | --- | --- |

**Supplementary Table 2. Basic characteristics of medical usage.** KM: Korean medicine.

| Category | | Total | | Standard Care | | Korean medicine | |
| --- | --- | --- | --- | --- | --- | --- | --- |
|  |  | No. of claims | Percentage | No. of claims | Percentage | No. of claims | Percentage |
| Type of visit | Outpatient | 112,492 | 84.45 | 78,174 | 79.23 | 34,318 | 99.38 |
|  | Inpatient | 20,711 | 15.55 | 20,496 | 20.77 | 215 | 0.62 |
| Medical institution | Tertiary hospital/general hospital/hospital | 55,322 | 41.53 | 55,168 | 55.91 | 154 | 0.45 |
|  | Clinic | 21,336 | 16.02 | 21,336 | 21.62 | - | - |
|  | Convalescent hospital | 21,582 | 16.2 | 21,162 | 21.45 | 420 | 1.22 |
|  | KM hospital | 4,131 | 3.1 | 1,004 | 1.02 | 3,127 | 9.06 |
|  | KM clinic | 30,832 | 23.15 | - | - | 30,832 | 89.28 |

**Supplementary Table 3. Categories by subtypes of I69 Sequelae of cerebrovascular disease.**

| Subtype | | | 2016 | | 2017 | | 2018 | |
| --- | --- | --- | --- | --- | --- | --- | --- | --- |
|  |  |  | No. of patients | Percent | No. of patients | Percent | No. of patients | Percent |
| Hemorrhage | I69.0 | Sequelae of subarachnoid hemorrhage | 948 | 13.43 | 827 | 12.85 | 758 | 12.54 |
|  | I69.1 | Sequelae of intracerebral hemorrhage |  |  |  |  |  |  |
|  | I69.2 | Sequelae of other nontraumatic intracranial hemorrhage |  |  |  |  |  |  |
| Infarction | I69.3 | Sequelae of cerebral infarction | 4386 | 62.15 | 4087 | 63.5 | 3775 | 62.44 |
| Others | I69.4 | Sequelae of stroke, not specified as hemorrhage or infarction | 493 | 6.99 | 414 | 6.43 | 405 | 6.7 |
| Unspecified | I69.8 | Sequelae of other and unspecified cerebrovascular diseases | 1230 | 17.43 | 1108 | 17.22 | 1108 | 18.33 |
|  | | | | | | | | |

**Supplementary Table 4. Annual rates of change of number of claims and costs per medical service category.** Service categories in KM do not include physical therapy, treatment and surgery, anesthesia, diagnostic radiology and radiotherapy, special equipment, and psychotherapy. All costs were converted using the annual average exchange rate (KRW/USD) of the corresponding year. The price level of healthcare cost is adjusted according to the health sector consumer price index for 2018 (see Supplementary Table 5). * p-value < 0.05; ** p-value < 0.01.

| Service category | Standard Care | | | | | | Korean Medicine | | | | | |
| --- | --- | --- | --- | --- | --- | --- | --- | --- | --- | --- | --- | --- |
|  | Average number of claims | Annual change rate (%) | Average annual claim per patient* | Annual change rate (%) | Average annual cost per patient* | Annual change rate (%) | Average number of claims | Annual change rate (%) | Average annual claim per patient* | Annual change rate (%) | Average annual cost per patient* | Annual change rate (%) |
| Consultation | 55,050 | -8.16 | 10.25 | -1.14 | $60.84 | 11.3 | 11,925 | -2.38 | 17.28 | -0.2 | $116.95 | 6.2 |
| Hospitalization | 31,494 | -17.67 | 23.25 | 2.17 | $2,431.61 | 4.9 | 422 | -7.23 | 11.16 | 15.21 | $1,482.69 | 34.81 |
| Prescription / medication | 30,875 | -25.52 | 19.00 | -10.48 | $191.22 | 1.1 | 4,953 | 4.03 | 24.62 | -3.24 | $22.34 | 15.52 |
| Injection / procedures | 16,533 | -23.23 | 14.61 | -10.76 | $174.43 | -13.14 | 40,120 | 0.63 | 58.38 | 3.26 | $228.27 | 11.89 |
| Anesthesia | 435 | -5.35 | 3.89 | 2.49 | $139.29 | 12.94 |  |  |  |  |  |  |
| Physical therapy | 31,778 | -17.51 | 39.07 | 1.28 | $2,537.00 | -6.83 | - | - |  |  | - | - |
| Treatment and surgery | 4,246 | -36.98 | 8.13 | -18.64 | $524.29 | -5.97 | - | - |  |  | - | - |
| Testing | 68,226 | -17.04 | 30.54 | -9.25* | $176.58 | -2.9 | 31 | -1.75 | 1.26 | -3.8 | $4.56 | -2.72 |
| Diagnostic radiology and radiotherapy | 4,584 | -20.12 | 4.55 | -7.85** | $53.42 | 5.22 | - | - |  |  | - | - |
| Special equipment | 1,534 | - | 2.22 | - | $166.65 | - | - | - |  |  | - | - |
| Psychotherapy | 183 | -1.68 | 2.99 | -1.68 | $54.20 | 16.84 | - | - |  |  | - | - |
| Others | 4,539 | - | 4.71 | - | $5,290.28 | - | - | - |  |  | - | - |

**Supplementary Table 5. ATC codes of relevant drug categories.** ATC: Anatomical Therapeutic Chemical.

| **Category** | **ATC code** |
| --- | --- |
| Hypotensors | C01A, C02C, C03A, C03C, C03D, C07A, C09A, C09C, C09D, C10B |
| Vasodilators | C01D, C01E, C04A, C08C, N07C |
| Antithrombotic agents | B01A, C04AD03 |
| Muscle relaxants | M01A, M02A, M03A, M03B, M03C, N02A, N02B |
| NSAIDs | N06B, N06D, N07A, C04AX |
| Psychiatric / neurological agents | N03A, N04B, N05A, N05B, N05C, N06A |
| Urologicals | G04B, G04C, N07AB02 |
| Digestives | A02A, A02B, A02X, A03, A03A, A03B, A03F, A04A, A05A, A06A, A07B, A07D, A16A |
| Hyperlipidemia drugs | C10A |
| Anti-diabetic drugs | A10A, A10B |
| Others | J01C, J01D, J01M, J02A, B05B, B05C, B05X, V07A, A05B, A11B, A11E, A11G, A13B, B03A, B03B, C05C, H02A, M09A, N01B, R01B, R03B, R03D, R05C, R05D, R05F, R06A, V03A |

**Supplementary Table 6. Annual average KRW-USD exchange rate and price level of healthcare cost.** This information is available on the following site: Korean Statistical Information Service (http://kosis.kr). The price level of healthcare cost is adjusted according to the health sector consumer price index for 2018.

| **Year** | **KRW/USD** | **Healthcare & medical service price index** |
| --- | --- | --- |
| 2016 | 1160.41 | 0.9918 |
| 2017 | 1130.48 | 1.0005 |
| **2018** | **1100.58** | **1.0000** |
